# When Salpingectomy Is Not Salpingectomy—Ipsilateral Recurrence of Tubal Pregnancy

**DOI:** 10.1155/2009/524864

**Published:** 2009-10-28

**Authors:** Simona Fischer, Marc J. N. C. Keirse

**Affiliations:** Department of Obstetrics, Gynaecology and Reproductive Medicine, Flinders Medical Centre, Flinders University, Bedford Park, SA 5042, Australia

## Abstract

Theoretically, total salpingectomy eliminates the risk of an ipsilateral tubal pregnancy. However, total salpingectomy is difficult to achieve using endoloops alone. We describe a situation where this resulted in an ipsilateral recurrence of tubal pregnancy which required emergency intervention and removal of the tubal remnants.

## 1. Introduction

Despite major advances in its early diagnosis and treatment, ectopic pregnancy continues to account for a significant number of maternal deaths. A World Health Organization analysis of maternal deaths showed ectopic pregnancy to be responsible for 4.9% of all and 6.1% of direct maternal deaths in developed countries [1]. With a frequency of 1% to 2% of pregnancies [2, 3], it is the main cause of maternal death in early pregnancy in countries where unsafe abortion has been eliminated. However, ipsilateral ectopic pregnancy after partial or total salpingectomy is a rare occurrence with less than a dozen cases reported in the English literature in the last 10 years [4–12].

## 2. Case Report

A woman, aged 28, gravida 4, para 0, presented at 5 weeks gestation with lower abdominal pain and mild vaginal bleeding. She had 2 terminations of pregnancy and her last pregnancy had been ectopic, managed laparoscopically by a left salpingectomy using endoloops. Transvaginal ultrasound showed an empty uterus, no evidence of ectopic pregnancy, and a moderate amount of peritoneal fluid. *β*-hCG was 500 IU/L. After 48 hours, *β*-hCG had increased to 695 IU/L. A further 3 days later, the *β*-hCG level was 1,300 IU/L which prompted referral to hospital for further investigation. Ultrasound showed a left adnexal ectopic pregnancy measuring 20 × 16 × 15 mm with a yolk sac, but no evidence of a fetal heart. As the woman was clinically stable, methotrexate was administered intramuscularly at 50 mg/m^2^, with follow-up of *β*-hCG levels. 

Four days after the methotrexate administration, *β*-hCG had risen to 2,200 IU/L and another dose of 50 mg/m^2^ was administered. Four days after the second dose of methotrexate and 15 days after the initial presentation, she attended the emergency department with severe lower abdominal pain, vaginal bleeding, and an acute abdomen. The *β*-hCG level at that time was 1,300 IU/L. Laparoscopy showed a significant haemoperitoneum and an ectopic pregnancy on the proximal segment of the left fallopian tube, which had its mid-portion missing. The right tube and ovaries were normal. Both remnants of the left tube were removed laparoscopically and haemostasis established. She recovered well and *β*-hCG levels were below the level of detection 3 weeks later. Histology confirmed the clinical findings.

## 3. Discussion

Ectopic pregnancies occurred in 1.1% of pregnancies and caused 10 maternal deaths between 2003 and 2005 in the UK, with no evidence of these having become less important as a cause of direct maternal death over the last 20 years [3]. 

In this case, it took 15 days between the initial presentation and the definitive emergency treatment. At her first referral to hospital, the patient fulfilled all criteria commonly considered as conditional for methotrexate treatment and she was followed up appropriately. However, the further evolution with an acute abdomen and the need for emergency intervention is reminiscent of the times before the advent of vaginal ultrasound and *β*-hCG quantitation. It is speculative whether she would have undergone an earlier planned intervention to remove the ectopic and remaining tubal segments, if it had been suspected that the ectopic was located in a remaining tubal segment. It is also speculative whether methotrexate treatment would have been used initially, if there had been awareness that the ectopic was located in a tubal remnant. Indeed, most gynaecologists would perceive this as a firm indication to remove the tubal remnants either now or later on. Certain is, though, that the presence of a tubal remnant was not suspected because of her history of a previous left salpingectomy. 

There is a great deal of variation in surgical treatments of tubal pregnancies [13]. Although there is general agreement that, where feasible, the laparoscopic approach is to be preferred over laparotomy, there is less agreement on the specific procedures. The main area of debate still centers on the relative merits of salpingectomy versus salpingostomy, in terms of subsequent fertility and ectopic recurrences. A multicentre randomized controlled trial is currently underway to address these issues [14]. 

There is an extensive literature on the use of endoloops in laparoscopic surgery most of it related to gastrointestinal procedures. In gynaecology, endoloops became popular mainly for laparoscopic tubal ligation, mimicking the classical open surgery Pomeroy approach, as an alternative to fallopian rings and clips [15]. With regard to ectopic pregnancies, which are mostly located in the fallopian tube and may not permit a substantial delay between diagnosis and treatment, the main attraction of the endoloop technique is that it can be used by persons with basic rather than advanced laparoscopic skills [16]. A randomised controlled trial, comparing the endoloop approach with conventional electrocautery in 102 patients, was recently reported from Malaysia [17]. There were benefits to the endoloop approach in terms of a shorter operating time and less postoperative pain, but other outcomes were basically similar. The paper provides no information on the completeness of the tubal resection, though, and no information on long-term outcomes. 

We are aware of only one report on the long-term outcome of the endoloop approach for treatment of ectopic pregnancy and this related specifically to cornual (i.e., interstitial) ectopic pregnancies [18]. Of 18 women treated with endoloop tied around the cornual area, 14 had a wish for a further pregnancy and 12 of them achieved a pregnancy. Nine resulted in a term birth, two ended in miscarriage, and one was an ectopic again, but this occurred in the contralateral tube [18]. Our search further revealed only one case, such as ours, in which it was clear that the ipsilateral tubal pregnancy occurred after endoloop treatment [6]. We do not know how many have occurred that were not reported. It is not known either how many endoloop procedures are performed. Therefore, one cannot estimate the frequency of this complication. 

Salpingectomy, while not necessarily eliminating all ipsilateral ectopics, certainly prevents a tubal recurrence on that side. However, our case illustrates that it is fallacious to assume that total salpingectomy is always as total as the word implies. In fact, it is inherently difficult to achieve a total salpingectomy when using nothing but endoloops. Such cases and others that leave a tubal remnant may well need to be considered as akin to salpingostomy in terms of the risk of recurrence. Whilst few generalisations can ever be made from a case report, it is important for clinicians to be aware of this inherent problem, especially as it is not the only consequence. Hydrosalpinges can develop in tubal stumps, resulting in decreased fertility and an occasional need for further surgical intervention. 

In conclusion, it is unwise to discount ipsilateral tubal pregnancies too quickly. Surgical variations in what is purported to be salpingectomy are sufficiently large and their consequences important enough for clinicians to remain vigilant.
